# Elevated baseline serum glutamate as a pharmacometabolomic biomarker for acamprosate treatment outcome in alcohol-dependent subjects

**DOI:** 10.1038/tp.2015.120

**Published:** 2015-08-18

**Authors:** H W Nam, V M Karpyak, D J Hinton, J R Geske, A M C Ho, M L Prieto, J M Biernacka, M A Frye, R M Weinshilboum, D-S Choi

**Affiliations:** 1 Department of Molecular Pharmacology and Experimental Therapeutics, Mayo Clinic College of Medicine, Rochester, MN, USA; 2 Department of Psychiatry and Psychology, Mayo Clinic College of Medicine, Rochester, MN, USA; 3 Neurobiology of Disease Program, Mayo Clinic College of Medicine, Rochester, MN, USA; 4 Department of Biomedical Statistics and Informatics, Mayo Clinic College of Medicine, Rochester, MN, USA

## Abstract

Acamprosate has been widely used since the Food and Drug Administration approved the medication for treatment of alcohol use disorders (AUDs) in 2004. Although the detailed molecular mechanism of acamprosate remains unclear, it has been largely known that acamprosate inhibits glutamate action in the brain. However, AUD is a complex and heterogeneous disorder. Thus, biomarkers are required to prescribe this medication to patients who will have the highest likelihood of responding positively. To identify pharmacometabolomic biomarkers of acamprosate response, we utilized serum samples from 120 alcohol-dependent subjects, including 71 responders (maintained continuous abstinence) and 49 non-responders (any alcohol use) during 12 weeks of acamprosate treatment. Notably, baseline serum glutamate levels were significantly higher in responders compared with non-responders. Importantly, serum glutamate levels of responders are normalized after acamprosate treatment, whereas there was no significant glutamate change in non-responders. Subsequent functional studies in animal models revealed that, in the absence of alcohol, acamprosate activates glutamine synthetase, which synthesizes glutamine from glutamate and ammonia. These results suggest that acamprosate reduces serum glutamate levels for those who have elevated baseline serum glutamate levels among responders. Taken together, our findings demonstrate that elevated baseline serum glutamate levels are a potential biomarker associated with positive acamprosate response, which is an important step towards development of a personalized approach to treatment for AUD.

## Introduction

Alcohol use disorders (AUDs) affect ~4–5% of the world population^1, 2^ and impose a major socio-economic burden on global mental health.^3^ An imbalance between excitatory and inhibitory neurotransmitter signaling cascades in the brain is a main cause of physical dependence in AUD.^4, 5, 6^

Acamprosate (Campral), a taurine analog approved for treatment of alcohol dependence, is known to increase the time to relapse owing to its ability to reduce glutamatergic imbalances in the brain.^7, 8, 9, 10^ A meta-analysis of 17 studies with 4087 individuals demonstrated that continuous abstinence rates are significantly higher in acamprosate-treated patients compared with placebo.^11^ Additional meta-analyses validate this finding favoring acamprosate as a therapeutic agent for the maintenance of abstinence over other Food and Drug Administration-approved medications for AUD.^12, 13^ However, the treatment outcome is not universal as it appears that acamprosate is mainly effective in a sub-population of patients with AUD.^14^ This sub-population of patients with AUD may differ from non-responders not only on their clinical characteristics, but also based on their biochemical status.^15^ Thus, discovery of biomarkers that are associated with a positive therapeutic response to acamprosate may be essential to prescribe acamprosate over other treatment options.^16, 17, 18^

Pharmacometabolomics, the analysis of metabolomic profiles in the study of drug effects, identifies metabolite signatures (both at baseline and after drug exposure) as potential biomarkers to predict treatment outcomes.^19, 20, 21^ The Pharmacometabolomics Research Network has initiated the use of metabotypes to help elucidate drug mechanisms of action of serotonin reuptake inhibitors, lithium, cocaine, ketamine and antipsychotics, understand side effects, develop novel medications and predict drug response, especially for psychiatric disorders.^19, 22^ From a prediction of the drug response standpoint, metabolomics has been specifically used to identify baseline (pretreatment) metabolite signatures that predict treatment response to sertraline in major depressive disorder^23, 24^ and to antipsychotics in schizophrenia,^25, 26^ as well as to identify differences in metabolite levels from bipolar patients who responded and did not respond to ketamine.^27^ Here, we used a targeted pharmacometabolomics approach to identify significant differences in baseline levels of serum metabolites between alcohol-dependent subjects who remained abstinent (responders) and those who relapsed (non-responders) during 12 weeks of acamprosate treatment in community-based treatment programs. We have demonstrated the potential usefulness of elevated serum glutamate levels as a predictor of acamprosate response. To the best of our knowledge, this is the first study describing use of pharmacometabolomics in treatment of subjects with AUD. Moreover, we have provided experimental evidence supporting a potential biological mechanism underlying the reduction in glutamate levels by acamprosate treatment.

## Materials and methods

### Study design, participants, assessments and outcome measures

**Figure 1:**
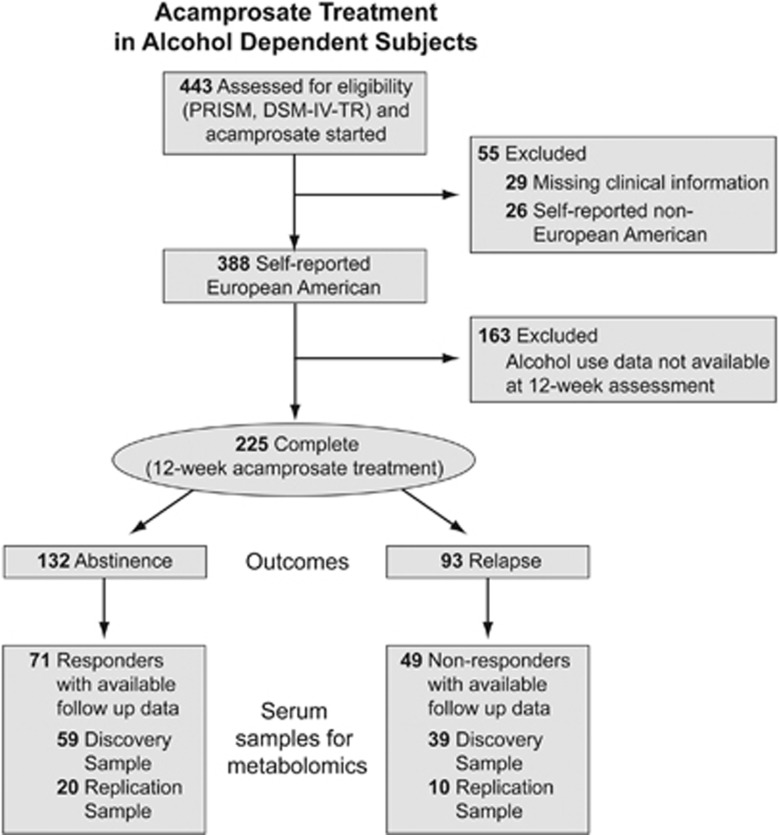
Consolidated Standards of Reporting Trials (CONSORT) diagram of the pharmacometabolomics biomarker study for acamprosate response in alcohol-dependent subjects. DSM-IV-TR, Diagnostic and Statistical Manual of Mental Disorders-4th Edition-Text Revision; PRISM, Psychiatric Research Interview for Substance and Mental Disorders.

This study was approved by the Institutional Review Board of Mayo Clinic Rochester and was conducted according to the Code of Ethics of the World Medical Association (Declaration of Helsinki). Study samples and clinical information were collected at baseline and during a 3-month follow-up visit from 120 subjects who provided informed consent as a subset of patients recruited as part of a previously described study.^28^ The process of sample selection is presented in Figure 1. Detailed description of the study participants, recruitment, assessments and study outcomes is presented elsewhere.^28^ In brief, all subjects met Diagnostic and Statistical Manual of Mental Disorders-4th Edition-Text Revision (DSM-IV-TR) criteria for alcohol dependence as determined by the semi-structured interview Psychiatric Research Interview for Substance and Mental Disorders.^29^ Subjects were recruited from community-based residential and outpatient treatment programs affiliated with Mayo Clinic in Rochester, Minnesota, and the Mayo Clinic Health System sites in Austin, Minnesota, Albert Lea, Minnesota and La Crosse, Wisconsin. In addition, a group of self-referred participants not enrolled in treatment but interested in taking acamprosate were recruited and included in the analyses as a separate study site. A description of programs and the number of subjects recruited at each site is presented in Supplementary Table 1.

All subjects were determined to be abstinent from alcohol for at least 5 days and no more than 6 months prior to the study entry (determined by timeline follow-back (TLFB) assessment) and to have no active signs of severe alcohol withdrawal (determined by Clinical Institute Withdrawal Assessment-Research assessment^30^). Subjects unable to provide informed consent; those unable to speak English; those with unstable medical or psychiatric conditions including moderate to severe renal or liver function impairment were excluded from the study; women who were pregnant, lactating or planning to become pregnant; subjects taking disulfiram; and those allergic to acamprosate were excluded from the study. Both self-reported history of drinking and sobriety information were collected via the TLFB^31^ assessment. In addition, both aspartate transaminase (AST) and gamma-glutamyl transpeptidase (GGT) levels were measured as a maker of sobriety before treatment.

All study participants received one 333-mg tablet three times a day for the first week to determine ability to tolerate medication. Then, a standard dose of two 333-mg tablets three times a day was prescribed. However, the research physician prescribing acamprosate to the study participants could also choose to allow participants to remain on one 333-mg tablet three times a day in order to reduce side effects and increase compliance in sensitive patients. Subjects were followed monthly using phone and in-person interviews conducted to obtain accurate sobriety, medication compliance and presence of psychiatric symptoms. The primary study outcome was defined as response (sobriety—that is, no alcohol use) versus non-response (any alcohol use) during 3 months of acamprosate treatment. The outcome was determined by self-report (TLFB). In addition, GGT levels were used to assess the accuracy of self-reported sobriety. The medication compliance rate was assessed through pill counting (percentage of pills taken to pills expected to be used) and blood acamprosate level using ultra-performance liquid chromatography tandem mass spectrometry (UPLC-MS/MS).

### Biospecimen collection and methods

A total of ~20 ml of blood was collected from each subject at baseline and at the 12-week follow-up visit. In the majority of cases, blood sample collection occurred between noon and 1500 hours; however, in some cases blood was drawn outside of this time period to accommodate individual subject's schedule. Venipuncture was performed using standard techniques. All tubes were labeled with a study identifier, collection date and time of draw. After collection, samples were electronically accessioned at the Biospecimens Accessioning Processing facility at Mayo Clinic. Samples were subsequently spun down for 15 min at 2900 *g* at 4 °C and serum was aliquoted into 250-μl samples and stored at −80 °C within 2 h to minimize any possible metabolite degradation. All serum samples were thawed on ice for ~2 h before use. Glutamine synthetase (GS) activity was measured as described.^32^

### Pharmacometabolomics using UPLC-MS/MS

Serum amino-acid calibration standards were prepared with MassTrak Amino Acid Analysis Solution (AAA) kit from Waters (Milford, MA, USA) according to instructions with slight modifications for detection on a mass spectrometer.^33^ A 10-point standard concentration curve was made from the calibration standard solution to calculate amino-acid concentrations in serum samples. Serum samples of 10 μl were spiked with an internal standard then derivatized according to MassTrak instructions. The amino-acid-derivatizing reagent used was 6-aminoquinolyl-*N*-hydroxysuccinimidyl carbamate. High-resolution separation was done using an Acquity UPLC system and injecting 1 ml of derviatized solution, with a UPLC BEH C18 column (Milford, MA, USA). Mass detection was completed on a TSQ Ultra Quantum running in ESI-positive mode (Thermo Scientific, Waltham, MA, USA).

### Assessment of the pharmacological effect of acamprosate on metabolite dynamics and liver GS activity in mice

Animal care and handling procedures were approved by the Mayo Clinic Institutional Animal Care and Use Committees in accordance with NIH guidelines. To measure acamprosate elimination and the metabolic response, acamprosate was administrated (intraperitoneally (i.p.); 200 mg kg^−1^) to C57BL/6 J mice (*n*=4 per treatment group), then 30 μl of blood was collected from the tail at 5 min, 30 min, 1 h and 2 h after treatment. Samples were centrifuged for 15 min at 2900 *g* at 4 °C and 10 μl of serum was collected and then analyzed using UPLC-MS/MS. To examine GS activity in response to acute acamprosate treatment, mice were either exposed to ethanol (i.p.; 3.2 g kg^−1^ once per day for 5 days) or received saline as a control (*n*=5 per treatment group). Mice liver lysates were collected as described.^34^ The liver lysates (20 μg) were treated with various concentrations of acamprosate [0 (0.9% NaCl), 1 μm, 10 μm, 100 μm and 1 mm] for 30 min. GS activity was measured as described.^32^

### Statistical analysis

Data are described as mean±s.d. for clinical data and mean±s.e.m. for metabolomics and preclinical data. Statistical analyses were performed using the Wilcoxon rank-sum test, paired Wilcoxon signed-rank test or linear regression (Prism v 5.04, GraphPad Software, La Jolla, CA, USA), SAS (version 9.3, Cary, NC, USA) and one-way or two-way analysis of variance (SigmaStat v. 3.1, SYSTAT Software, Point Richmond, CA, USA). Results were considered nominally significantly different when *P*<0.05.

## Results

### Clinical characteristics of study participants

To investigate biomarkers of acamprosate treatment outcome, we used data from 120 alcohol-dependent subjects (DSM-IV-TR) from community-based treatment programs affiliated with Mayo Clinic (Figure 1). Clinical characteristics were assessed and serum samples were collected at baseline and after 12 weeks of acamprosate treatment. Treatment outcome (abstinence or relapse) was assessed monthly using TLFB.^31^ Positive treatment outcome was defined by abstinence (no alcohol use per TLFB), and GGT levels were used to assess the accuracy of self-reported sobriety at the 12-week follow-up visit.

**Figure 2:**
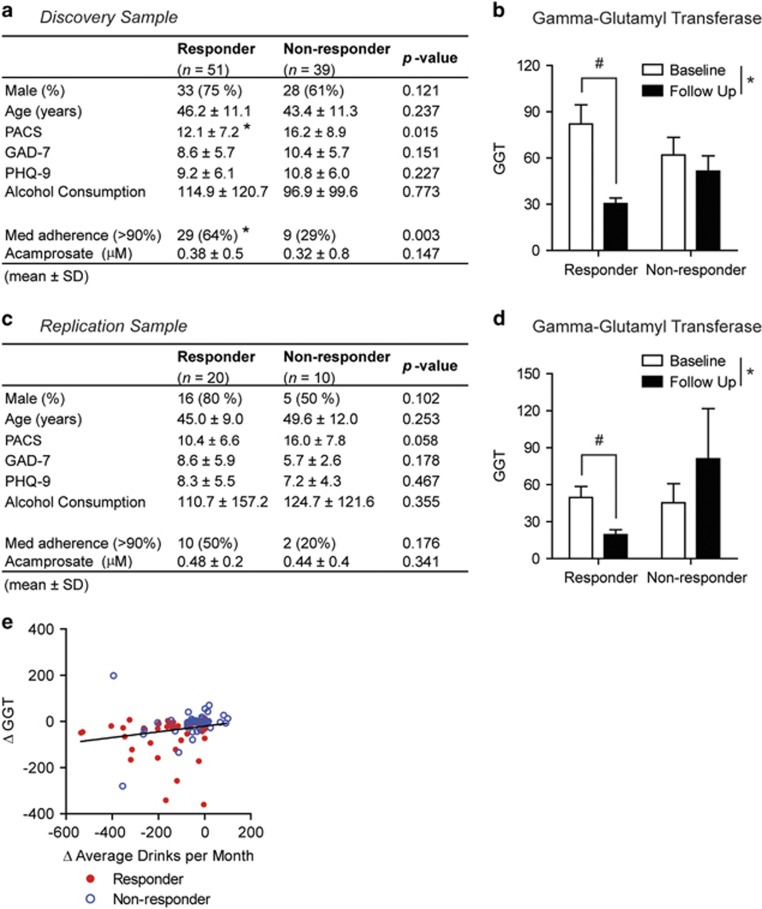
Clinical characteristics of acamprosate responders. (**a**) Baseline demographics of the discovery cohort (90 subjects). PACS, GAD-7 scale, PHQ-9 and TLFB-30 assessment were used to assess craving, anxiety, depression and alcohol consumption, respectively. Data are reported as mean±s.d. *P<0.05 by the Wilcoxon rank-sum or *χ*^2^-test. (**b**) GGT plasma levels in responders and non-responders to acamprosate treatment in the discovery cohort. In the responder group, GGT levels returned back to a normal range after acamprosate treatment, whereas GGT levels remained elevated in the non-responder group. Data are reported as mean±s.e.m. Statistics is by two-way ANOVA followed by the Tukey *post hoc* test. *P<0.05 for main effect of time. ^#^*P*<0.05 for individual comparisons. (**c**) Baseline demographics of the replication cohort (30 subjects). The PACS, GAD-7, PHQ-9 and TLFB-30 were used to assess craving, anxiety, depression and alcohol consumption, respectively. Data are reported as mean±s.d. (**d**) GGT plasma levels in responders and non-responders to acamprosate treatment in the replication cohort. In the responder group, GGT levels returned back to a normal range after acamprosate treatment, whereas GGT levels remained elevated in the non-responder group. Data are reported as mean±s.e.m. Statistics is by two-way ANOVA followed by the Tukey *post hoc* test. *P<0.05 for main effect of time. ^#^*P*<0.05 for individual comparisons. (**e**) A change in TLFB-30 (follow-up−baseline) and change in plasma GGT levels (follow-up−baseline) were significantly correlated (*n*=101). *P*<0.05 by Spearman correlation analysis. ANOVA, analysis of variance; GAD-7, Generalized Anxiety Disorder 7-item scale; GGT, gamma-glutamyl transpeptidase; PACS, Pennsylvania Alcohol Craving Scale; PHQ-9, 9-item The Patient Health Questionnaire; TLFB-30, 30-day timeline follow-back assessment.

Clinical characteristics of subjects included in each cohort and their association with outcomes to acamprosate treatment are presented in Figures 2a and c. In the discovery cohort, the responder (*n*=51) and non-responder (*n*=39) groups did not differ significantly in age, gender, alcohol consumption (measured by 30-day TLFB total drinks in the past 30 days; TLFB-30) or intensity of depressive (measured using the Patient Health Questionnaire scale, PHQ-9) or anxiety (measured using the Generalized Anxiety Disorder scale, GAD-7) symptoms. As expected, responders and non-responders that were diagnosed with depression had significantly increased PHQ-9 scores compared with subjects that did not have a depression diagnosis (Supplementary Figure 1a). Craving intensity measured by the Pennsylvania Alcohol Craving Scale^35^ was significantly lower in acamprosate responders compared with the non-responder group (Figure 2a). Baseline GGT levels were elevated in both responders and non-responders but returned to a normal range after 12 weeks of acamprosate treatment only in responders (Figure 2b). Although the medication adherence was higher in responders than non-responders, serum acamprosate levels (measured at a 12-week follow-up visit) were similar between the groups (Figure 2a).

In the replication sample, the responder and non-responder groups did not differ significantly in age, gender, craving alcohol consumption and intensity of depression or anxiety symptoms (Figure 2c). Consistent with results in the discovery sample, in the replication sample, GGT levels returned to a normal range following 12 weeks of acamprosate treatment in the responder group (Figure 2d).

In addition, we analyzed a correlation between GGT plasma levels and alcohol consumption (TLFB-30 average number of drinks) in 101 subjects (57 responders and 44 non-responders). To determine whether self-reported drinking (TLFB-30) was a reliable measure of treatment response, we examined whether there was a correlation between a change in self-reported alcohol consumption and a change in plasma GGT levels. We found that there was a significant correlation between a change in TLFB-30 (follow-up−baseline) and change in plasma GGT levels (follow-up−baseline) in the total sample of 101 subjects (Spearman's *r*=0.43; *P*<0.0001; Figure 2e). In addition, we found a significant correlation between a change in TLFB-30 (follow-up−baseline) and change in plasma GGT levels (follow-up−baseline) in responders (Spearman's *r*=0.33; *P*=0.013) and in non-responders (Spearman's *r*=0.39; *P*=0.009).

### Increased baseline serum glutamate level in acamprosate responders

**Figure 3:**
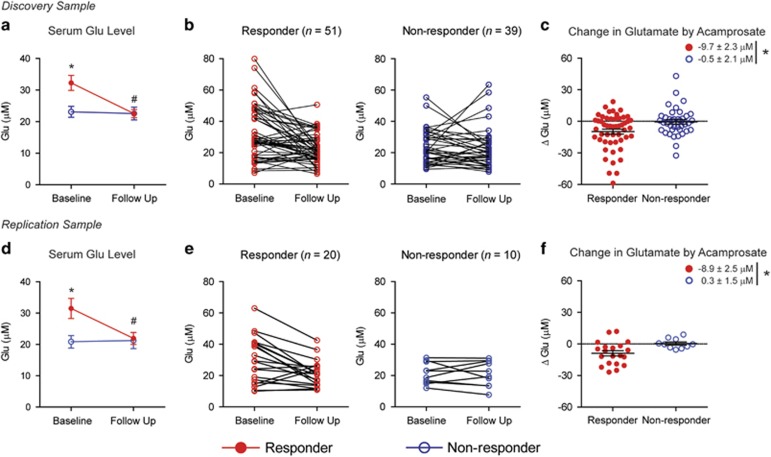
Serum glutamate levels at baseline and after treatment in acamprosate responders and non-responders. In the discovery cohort (*n*=90; *n*=51 for the responder group and *n*=39 for the non-responder group): (**a**) comparison of the average glutamate levels in the discovery cohort. The baseline glutamate level was significantly higher in the responder group compared with the non-responder group (Wilcoxon rank-sum test, *P=0.012), whereas no between-group difference in glutamate levels was observed at 3 months follow-up. In the responder group, serum glutamate levels decreased significantly (paired Wilcoxon signed-rank test, ^#^*P*<0.001) after 12 weeks of acamprosate treatment. We observed no significant change in glutamate levels between baseline and follow-up in the non-responder group. Data are reported as mean±s.e.m. (**b**) Individual profiles reflecting changes in serum glutamate levels in acamprosate treatment responders and non-responders. (**c**) Comparison of changes in serum glutamate levels in the responder and non-responder groups. Individual changes are presented by colored dots. The responder group showed significantly decreased serum glutamate by acamprosate treatment, whereas the non-responder group did not (Wilcoxon rank-sum test, *P=0.031). Data are reported as mean±s.e.m. In the replication study (*n*=30; *n*=20 for the responder group and *n*=10 for the non-responder group): (**d**) comparison of the average glutamate levels in the replication cohort. The baseline glutamate level was significantly higher in the responder group compared with the non-responder group (Wilcoxon rank-sum test, *P=0.036), whereas no between-group difference in glutamate levels was observed at 3-month follow-up. In the responder group, serum glutamate levels decreased significantly (paired Wilcoxon signed-rank test, ^#^*P*=0.001) after 12 weeks of acamprosate treatment. We observed no significant change in glutamate levels between baseline and follow-up in the non-responder group. Data are reported as mean±s.e.m. (**e**) Individual profiles reflecting changes in serum glutamate levels in acamprosate treatment responders and non-responders. (**f**) Comparison of changes in serum glutamate levels in the responder and non-responder groups. Individual changes are presented by colored dots. The responder group showed significantly decreased serum glutamate by acamprosate, whereas the non-responder group did not (Wilcoxon rank-sum test, *P=0.014). Data are reported as mean±s.e.m.

As acamprosate is an amino-acid derivative and is known to reduce glutamate levels in the brain,^7, 8, 9, 10^ we hypothesize that acamprosate may influence homeostasis of glutamate and other amino acids or their derivatives in the blood. Thus, we profiled 36 metabolites including 20 amino acids and acamprosate at baseline and in response to acamprosate treatment using UPLC-MS/MS.^33^ Each metabolite measured at baseline and after 12 weeks of acamprosate treatment is presented in Supplementary Table 2. In the discovery cohort, 14 metabolites showed nominally significant differences between baseline and follow-up levels (*P*<0.05). In the replication sample, however, only four metabolites showed nominally significant differences between baseline and follow-up levels in a direction similar to the discovery cohort (Supplementary Table 2). Notably, as shown in Figures 3a and b, in the discovery sample, glutamate levels were significantly higher at baseline in responders (32.3±2.4 μm) compared with non-responders (23.1±1.7 μm; *P*=0.012). Following acamprosate treatment, serum glutamate levels in the responder group decreased significantly (−9.7±2.3 μm) compared with baseline (*P*<0.001), whereas non-responders showed no change in glutamate level (−0.5±2.1 μm; Figure 3c). Consistently, in the replication sample, glutamate levels were elevated at baseline in responders (31.5±3.2 μm) compared with non-responders (20.8±2.0 μm; *P*=0.036; Figures 3d and e). Following 12 weeks of acamprosate treatment, glutamate levels in the responder group decreased significantly (−8.9±2.5 μm) compared with baseline (*P*=0.001), whereas non-responders showed no change (0.3±1.5 μm; Figure 3f). In addition, we did not find that the presence or absence of a depression diagnosis impacted the level of baseline glutamate levels. Thus, regardless of whether or not a subject was diagnosed with depression, responders to acamprosate exhibited significantly increased glutamate levels compared with non-responders (Supplementary Figure 1b).

In addition, sensitivity analyses for the primary results in the discovery sample were performed to assess potential confounding or bias due to differences between five recruitment sites, as well as batch effect (metabolomics assays in the discovery group were performed in two batches). Multivariable logistic regression models were used to evaluate the association of glutamate and ammonia levels with treatment response while accounting for these covariates. This analysis demonstrated that higher baseline glutamate (odds ratio (OR)=1.07, *P*=0.004) or ammonia (OR=1.07, *P*=0.023) were predictors of response (OR=1.07, *P*=0.023) after adjusting for batch and site in the discovery sample.

### Potential role of GS in the pharmacological effect of acamprosate

**Figure 4:**
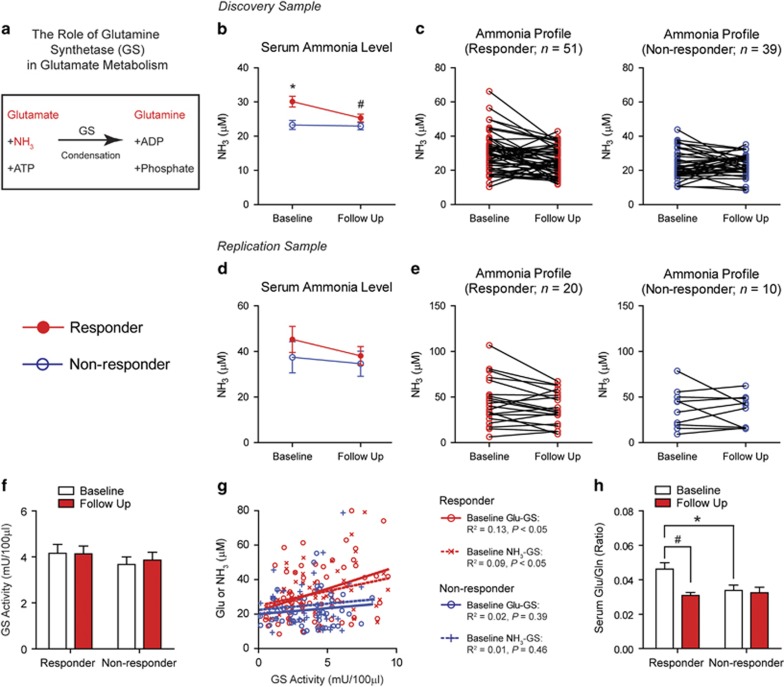
Acamprosate responders showed activated GS metabolism. (**a**) Glutamate is metabolized by GS by condensation with ammonia to form glutamine. In the discovery cohort (*n*=90; *n*=51 for the responder group and *n*=39 for the non-responder group). (**b**) the baseline ammonia level was significantly higher in the responder group compared with the non-responder group (Wilcoxon rank-sum test, *P=0.003), whereas no between-group difference in glutamate levels was observed between the responder and non-responder groups at 3 months follow-up. In the responder group, serum ammonia levels decreased significantly (paired Wilcoxon signed-rank test, ^#^*P*=0.002) after 12 weeks of acamprosate treatment. We observed no significant change in ammonia levels between baseline and follow-up in the non-responder group. (**c**) Individual profiles reflecting changes in serum ammonia levels following acamprosate treatment in responders and non-responders in the discovery cohort. In the replication cohort (*n*=30; *n*=20 for the responder group and *n*=10 for the non-responder group). (**d**) The baseline ammonia level did not differ from the non-responder group; however, there was a trend for it to be higher. No difference in glutamate levels was observed between the responder and non-responder groups at 3 months follow-up. In the responder group, serum ammonia levels tended to decrease after 12 weeks of acamprosate treatment, although it was not significant. We observed no significant change in ammonia levels between baseline and follow-up in the non-responder group. (**e**) Individual profiles reflecting changes in serum ammonia levels following acamprosate treatment in responders and non-responders in the replication cohort. (**f**) Serum GS activity in responders and non-responders in the combined sample (*n*=71 for the responder group and *n*=49 for the non-responder group; the combined sample). Serum GS activity showed no significant change in response to acamprosate treatment or between responders and non-responders. Data are reported as mean±s.e.m. (**g**) Correlation between GS activity and glutamate or ammonia levels in responders and non-responders in the combined sample (*n*=71 for the responder group and *n*=49 for the non-responder group; the combined sample). Analyses indicate a significant positive correlation (linear regression analysis) between GS activity and baseline glutamate or ammonia levels in the responder group, whereas no such correlation was observed in the non-responder group. (**h**) Glutamate/glutamine ratio in the responder and non-responder groups in response to acamprosate in the combined sample (*n*=71 for the responder group and *n*=49 for the non-responder group; the combined sample). The responder group showed a significantly increased glutamate/glutamine ratio at baseline compared with the non-responder group (Wilcoxon rank-sum test, *P=0.016). This elevated glutamate/glutamine ratio decreased after acamprosate treatment in the responder group (paired Wilcoxon signed-rank test, ^*#*^*P*<0.0001), whereas no change was observed in non-responders. Data are reported as mean±s.e.m. GS, glutamine synthetase.

To investigate a potential mechanism for the pharmacological effect of acamprosate, we examined metabolic pathways for metabolites showing a significant difference in responders and non-responders. Interestingly, in the discovery cohort, we found that ammonia levels were increased in baseline and reduced upon acamprosate treatment in responders similar to glutamate levels (Figures 4b and c), suggesting that that glutamate–ammonia condensation by GS has an essential role in the pharmacological effect of acamprosate (Figure 4a).^36^ In the replication cohort, although we were unable to replicate this finding possibly due to small sample numbers, there is a trend similar to the discovery cohort (Figures 4d and e). We did not find that the presence or absence of a depression diagnosis impacted the level of baseline ammonia levels. Thus, regardless of whether or not a subject was diagnosed with depression, responders to acamprosate exhibited significantly increased ammonia levels compared with non-responders (Supplementary Figure 1c).

Next, we examined serum GS activity in responders and non-responders. Despite the fact that there was no difference in averaged GS activity between responders and non-responders or between before and after acamprosate treatment (Figure 4f), we found that only responders showed significant correlations between basal GS activity and glutamate or ammonia levels (Figure 4g). This result strongly suggests that elevated baseline glutamate and/or ammonia levels increase GS activity in responders. Then, we investigated the glutamate/glutamine ratio as a potential indicator of GS activity. Consistently, the baseline serum glutamate/glutamine ratio was significantly increased in the responder group compared with the non-responder group (Figure 4h). Furthermore, responders showed a significant reduction in the glutamate/glutamine ratio after acamprosate treatment, whereas the non-responders group showed no differences after acamprosate treatment (Figure 4h).

### Elevated GS activity by acamprosate in mice

**Figure 5:**
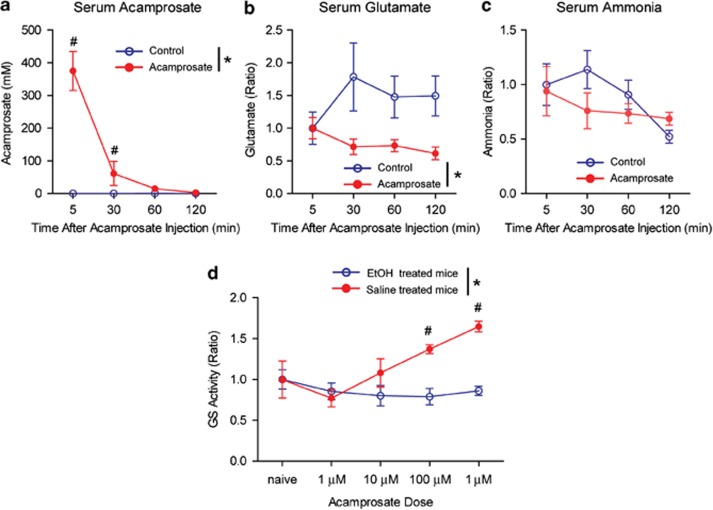
Acamprosate treatment increased GS metabolism in mice. (**a**) Acute acamprosate treatment (i.p., 200 mg kg^−1^) was eliminated 60 min after acute acamprosate administration to alcohol-naive mice (*n*=4). Two-way ANOVA followed by the Tukey *post hoc* test indicated that the serum acamprosate level was no longer detectable 60 min after the acamprosate injection relative to a saline-injected control cohort of mice (*n*=4). *P<0.05 for main effect of treatment. ^#^*P*<0.05 for individual comparisons. Data are reported as mean±s.e.m. (**b**) Following acute acamprosate administration (i.p., 200 mg kg^−1^) glutamate was significantly reduced. Two-way ANOVA followed by the Tukey *post hoc* test indicated a significant effect of treatment, whereas there was no effect of time or an interaction between treatment and time. *P<0.05 for main effect of treatment. Data are reported as mean±s.e.m. (**c**) Following acute acamprosate administration (i.p., 200 mg kg^−1^) ammonia levels were not significantly altered. Data are reported as mean±s.e.m. (**d**) Acamprosate incubation for 30 min increased GS activity in mouse liver lysate. Two-way ANOVA indicated that chronic ethanol treatment increased GS activity. A significant main effect of treatment and acamprosate concentration as well as an interaction were detected (*F*_(1,48)_=4.8, *P*<0.05 for the interaction). The Tukey *post hoc* test revealed that acamprosate increased GS activity at the 10 μm (*P*=0.001) and 1 mm (*P*<0.001) concentrations. One-way ANOVA for saline-treated liver lysate indicated a significant effect of acamprosate dose (*F*_(4,19)_=5.9, *P*<0.05), and the Tukey *post hoc* test revealed significantly increased GS activity by 1 mm acamprosate treatment (*P*<0.05) compared with naive control (*n*=5). *P<0.05 for main effect of treatment and ^#^*P*<0.05 for individual comparisons by the Tukey *post hoc* test. Data are reported as mean±s.e.m. ANOVA, analysis of variance; GS, glutamine synthetase; i.p., intraperitoneally.

To investigate whether acamprosate promotes GS activity, we sought to quantify GS activity in response to acamprosate in mice. First, we examined blood acamprosate levels upon acute acamprosate administration (i.p., 200 mg kg^−1^) to alcohol-naive C57BL/6 J mice. We found that the majority of acamprosate was eliminated from the serum within 60 min (Figure 5a). Interestingly, serum glutamate levels were significantly reduced in acamprosate-treated mice compared with saline-treated control mice (Figure 5b). Although ammonia levels were not significantly altered, there was a trend (Figure 5c) similar to clinical studies (Figure 4). Finally, we examined GS activity in the liver lysate where GS is abundantly active^37^ in response to acute acamprosate treatment from both saline-treated control mice and mice exposed to chronic ethanol (i.p., 3.2 g kg^−1^ once per day for 5 days). We found that liver GS activity is significantly increased in response to acamprosate in a dose-dependent manner in saline-treated control mice. Whereas there were no changes in GS activity in ethanol-treated mice (Figure 5d), indicating that acamprosate promotes GS activity in liver lysate only in the absence of ethanol.

## Discussion

Our present study provides a potential serum metabolomic biomarker that is associated with pharmacological response to acamprosate in alcohol-dependent subjects. Our main findings demonstrated that baseline serum glutamate levels were significantly higher in acamprosate responders compared with non-responders. Notably, in responders, glutamate levels were significantly decreased after 12 weeks of acamprosate treatment relative to baseline, whereas there was no change in glutamate levels in non-responders after the same length of acamprosate treatment. In addition, the pattern of serum ammonia levels was parallel to glutamate levels, implying that GS has an essential role in the pharmacological effect of acamprosate. Together with functional studies in mouse models, our results suggest that acamprosate treatment promotes GS activity, which may be a potential mechanism explaining the reduction of glutamate levels by acamprosate in responders.

Overall, our study revealed that serum metabolites (for example, glutamate and ammonia) differentiate responders and non-responders to acamprosate. Similar pharmacometabolomic approaches have been successfully used to identify metabolic signatures of neuropsychiatric and other diseases.^19, 38^ Importantly, the UPLC-MS/MS-based detection of baseline metabolic signatures could be easily implemented in a clinical setting,^39^ which makes translation of these findings into clinical practice feasible. Therefore, prospective studies using glutamate and/or ammonia levels for selection of potential responders to acamprosate are necessary to further validate the usefulness of metabolomic biomarkers in pharmacotherapy of AUD.

It is important to emphasize that investigation of metabolic signatures in our study was restricted to serum, where the majority of metabolites are metabolized by liver enzymes. Although useful as potential biomarkers of treatment response, it is not known at this point if similar metabolic signatures reflect the effects of acamprosate in the brain. However, evidence indicates similarities in the relationship between glutamate and glutamine levels in blood and the brain through efflux of glutamate.^40^ In particular, blood glutamate scavengers increase the efflux of glutamate from the brain to the blood.^41^ Thus, it is possible that reduction of serum glutamate levels is due to activating effects of acamprosate on GS in the liver. In addition, a reduction of the serum glutamate/glutamine ratio in response to acamprosate is consistent with a reduction in glutamate levels in response to acamprosate observed in both human and rodent brains measured by magnetic resonance spectroscopy.^9, 10^ As GS is highly expressed in astrocytes,^36^ our findings suggest that GS-related metabolism could also be essential to the pharmacological effect of acamprosate in the brain.^8, 42^

In addition to the presented findings, several of the metabolites we detected are involved in the urea cycle, indicating that malfunction in ammonia elimination may be among the potential pathways for further experimentation in relation to elevated serum glutamate levels in acamprosate responders. It is also important to consider that ammonia can cross the blood–brain barrier and has been shown to result in increased glutamate levels in the brain.^43^ As glutamate dehydrogenase (GLDH) and GS have an important role in signaling regulation in alcohol dependence,^44^ we also analyzed their metabolic products. GLDH converts glutamate to α-ketoglutarate releasing ammonia and vice versa, whereas GS condenses glutamate and ammonia to glutamine. Although GLDH has been suggested to be a potential alcohol dependence biomarker,^44^ we found that GS activity could be an underlying mechanism for positive acamprosate treatment outcome as glutamine was negatively correlated with both glutamate and ammonia in the responder group. It is important to note that acamprosate is most effective in maintaining abstinence when patients were detoxified before initiating acamprosate treatment.^45^ It is also possible that alcohol interferes with GS activity and diminishes the pharmacological effect of acamprosate. Therefore, further investigation is warranted into the role of GS in response to acamprosate and in ethanol-related behaviors.

Our findings should be considered in the context of the following limitations. First, our study focused on identifying biomarkers associated with abstinence in acamprosate-treated alcoholics and did not include a placebo control group, or complete follow-up of all patients in the treatment group. Therefore, study samples collected in our discovery and replication cohorts do not allow analyses separating acamprosate-specific effects from effects associated with other factors contributing to abstinence. Thus, we primarily focused on baseline levels of metabolites to identify metabolomic biomarkers associated with acamprosate treatment response. Moreover, we were unable to completely control for other possible confounding factors that could impact levels of amino acids and their derivatives including use of other medications, diet and circadian rhythms, which should be considered as a possible limitation of our study. The second potential limitation is that we have only investigated amino acids and their derivatives. Future studies using unbiased global metabolomics will reveal additional biomarkers associated with acamprosate response such as lipids or carbohydrates, which are implicated in psychiatric disorders.^46, 47^ In conclusion, our study demonstrates the usefulness of pharmacometabolomic biomarkers for a personalized approach in treating AUD.

## Notes

The medication (acamprosate) used in this study was in part provided by Forest Pharmaceuticals. Other than providing medication, neither Forest Pharmaceuticals nor its affiliates were involved in study conceptualization, design, data collection, data analysis, interpretation or preparation of the manuscript. Dr MAF has had grant support from Pfizer and Myriad, and has served as an unpaid consultant for Allergan, Myriad, Sunovion and Teva Pharmaceuticals. The remaining authors declare no conflict of interest.
