# Supplementary material for: Elevated baseline serum glutamate as a pharmacometabolomic biomarker for acamprosate treatment outcome in alcohol-dependent subjects
Source: Transl Psychiatry. 2015 Aug 18;5(8):e621–. doi: 10.1038/tp.2015.120 (PMC4564571; doi:10.1038/tp.2015.120)
Supplement: Supplementary Information [file tp2015120x1.pdf]

Supplemental Table 1: Subject Recruitment Per Study Site.

Study Site	Discovery Sample				Replication Sample			
	Relapse	Non-relapse	Total	Percent	Relapse	Non-relapse	Total	Percent
Mayo Clinic Rochester, MN	18	7	25	28	10	2	12	40
Albert Lea, MN	20	7	27	30	6	1	7	23
Austin, MN	5	13	18	20	1	3	4	13
La Crosse, WI	3	4	7	8	2	3	5	17
Self-Referral	5	8	13	14	1	1	2	7
Total	51	39	90	100	20	10	30	100

Supplemental Table 2: Pharmacometabolomics profile measured by LC-MS/MS.

Metabolites	Discovery Sample				Replication Sample			
	Responder (n = 51)		Non-Responder (n = 39)		Responder (n = 20)		Non-Responder (n = 10)	
	Baseline	Follow Up	Baseline	Follow Up	Baseline	Follow Up	Baseline	Follow Up
3-Methylhistidine	5.19 ± 2.90	4.78 ± 2.25	4.87 ± 2.04	4.80± 1.97	2.13 ± 1.47	2.83 ± 1.70	2.78 ± 2.12	2.79 ± 1.34
α-Aminoadipic-acid	0.85 ± 0.58 *	0.77 ± 0.54	0.61 ± 0.41	0.62± 0.38	1.73 ± 0.48	1.34 ± 0.49	1.66 ± 0.52	1.34 ± 0.49
α-Amino-N-butyric-acid	14.14 ± 5.45	14.23 ± 5.85	14.50 ± 6.19	15.06 ± 7.20	12.34 ± 4.89	13.21 ± 5.06	19.76 ± 8.77	15.78 ± 8.31
Alanine	400.40 ± 69.83	411.90 ± 102.60	392.90 ± 98.58	420.50 ± 91.13	400.2 ± 102.5	412.00 ± 81.74	378.30 ± 68.98	408.60 ± 88.50
Ammonia	30.12 ± 11.11*	25.33 ± 8.08 [#]	23.28 ± 8.29	26.29 ± 21.86	45.33 ± 25.49	38.13 ± 18.15 [#]	37.46 ± 21.53	34.66 ± 17.41
Arginine	104.20 ± 23.25	105.30 ± 27.85	100.70 ± 24.80	99.63 ± 22.92	90.65 ± 21.80	95.64 ± 28.00	89.42 ± 23.29	85.07 ± 24.56
Asparagine	57.46 ± 13.12	60.34 ± 15.90	58.02 ± 16.46	57.99 ± 13.68	70.73 ± 15.46	74.27 ± 14.73	76.83 ± 13.34	69.78 ± 17.59
Aspartate	21.03 ± 10.73 *	13.68 ± 6.97 [#]	13.61 ± 7.21	12.15 ± 6.61	13.82 ± 6.05	9.69 ± 5.07 [#]	10.60 ± 5.12	10.06 ± 4.91
β-Alanine	5.42 ± 2.11	6.41 ± 6.48	6.66 ± 5.68	6.40 ± 5.26	5.56 ± 1.92	7.47 ± 7.47	4.85 ± 1.89	4.31 ± 2.18
Citrulline	28.17 ± 8.78	30.89 ± 9.60 [#]	30.36 ± 9.95	30.30 ± 9.21	27.07 ± 6.40	27.53 ± 5.62	27.63 ± 7.86	30.56 ± 10.36
Cystathionine	0.47 ± 0.41	0.44 ± 0.48 [#]	0.46 ± 0.47	0.32 ± 0.27	2.58 ± 0.44	2.60 ± 0.42	2.55 ± 0.38	2.58 ± 0.58
Cystine	75.84 ± 21.71	88.89 ± 25.83 [#]	78.84 ± 19.00	84.99 ± 28.05	82.22 ± 20.72	89.63 ± 20.73	89.58 ± 25.86	86.24 ± 29.89
Ethanolamine	8.25 ± 2.14 *	7.43 ± 1.89 [#]	7.27 ± 2.61	6.97 ± 2.06	10.01 ± 2.57	8.99 ± 3.21	10.53 ± 2.42	9.73 ± 2.20
GABA	0.11 ± 0.04	0.11 ± 0.04	0.12 ± 0.004	0.13 ± 0.07	0.13 ± 0.21	0.14 ± 0.22	0.21 ± 0.27	0.20 ± 0.26
Glutamate	32.29 ± 16.90 *	22.55 ± 9.33 [#]	23.13 ± 10.75	22.60 ± 12.70	31.47 ± 14.02 *	21.90 ± 8.24 [#]	20.84 ± 6.27	21.22 ± 8.09
Glutamine	715.70± 153.10	741.40 ± 134.30	714.70 ± 180.70	724.80 ± 144.80	809.00 ± 152.40	863.60 ± 124.00	936.90 ± 222.80	886.10 ± 181.60
Glycine	282.50 ± 101.20	292.10 ± 85.32	255.30 ± 65.54	266.00 ± 89.50	285.60 ± 70.72	309.70 ± 73.60	284.50 ± 51.28	287.90 ± 71.27
Histidine	91.52 ± 27.30	89.94 ± 23.41	90.12 ± 18.47	90.54 ± 16.61	88.74 ± 22.62	93.69 ± 21.71	94.97 ± 24.30	82.09 ± 18.59
Homocystine	0.28 ± 0.14	0.30 ± 0.17	0.23 ± 0.19	0.22 ± 0.17	1.17 ± 0.34	1.15 ± 0.34	1.26 ± 0.53	1.08 ± 0.37
Hydroxylysine	0.92 ± 0.65	0.86 ± 0.64	0.82 ± 0.55	0.83 ± 0.610	2.43 ± 0.50	2.30 ± 0.61	2.33 ± 0.52	2.32 ± 0.50
Hydroxyproline	16.57 ± 7.45	15.87 ± 8.19	14.65 ± 8.52	14.37 ± 7.70	18.31 ± 10.00	18.87 ± 7.80	16.34 ± 9.25	14.92 ± 7.20
Isoleucine	66.96 ± 23.15	64.61 ± 24.76	64.47 ± 21.36	61.91 ± 19.36	59.03 ± 15.44	68.90 ± 24.21	72.07 ± 23.91	53.16 ± 11.20
Leucine	139.60 ± 42.61	130.20 ± 42.06	130.70 ± 39.70	123.10 ± 35.02	113.40 ± 27.57	128.10 ± 39.75	130.90 ± 35.07	94.95 ± 14.34
Lysine	149.30 ± 42.75	149.80 ± 40.28	146.80 ± 40.98	138.90 ± 39.41	162.70 ± 50.44	154.10 ± 40.21	200.90 ± 99.37	159.5 ± 53.50
Methionine	20.51 ± 5.85	20.88 ± 6.80	21.23 ± 8.11	21.18 ± 6.83	19.51 ± 5.81	22.38 ± 8.05	21.91 ± 4.45	19.40 ± 3.90
Ornithine	100.70 ± 32.09 *	96.20 ± 26.08	82.18 ± 33.28	81.48 ± 27.69	78.37 ± 47.57	74.98 ± 32.86	88.12 ± 55.50	80.11 ± 40.58
Phenylalanine	73.98 ± 17.05 *	66.82 ± 13.90 [#]	66.66 ± 15.92	65.05 ± 14.10	59.77 ± 15.34	63.17 ± 17.14	62.78 ± 11.84	60.39 ± 9.86
Phosphoethanolamine	1.56 ± 1.09	1.12 ± 0.75 [#]	1.16 ± 0.69	0.99 ± 0.51	3.45 ± 2.63	2.57 ± 1.96	2.52 ± 2.15	1.72 ± 1.02
Proline	218.40 ± 64.57	212.40 ± 68.78	225.70 ± 86.84	232.80 ± 75.13	226.90 ± 57.27	242.40 ± 60.27	207.10 ± 48.84	230.00 ± 73.48
Sarcosine	1.01 ± 0.43	1.01 ± 0.65	1.04 ± 0.51	1.09 ± 0.53	1.65 ± 0.56	1.75 ± 0.61	2.13 ± 1.08	2.36 ± 2.50
Serine	112.40 ± 28.85	107.10 ± 22.71	103.20 ± 28.08	104.7 ± 27.08	115.10 ± 20.15	118.10 ± 27.55	127.00 ± 23.94	114.30 ± 30.07
Taurine	174.2 ± 96.53 *	141.1 ± 68.08 [#]	129.30 ± 50.63	125.30 ± 46.96	165.20 ± 90.80	132.30 ± 65.38	137.70 ± 57.92	126.70 ± 59.58
Threonine	114.80 ± 25.34	126.80 ± 34.77 [#]	129.00 ± 45.68	128.20 ± 41.93	110.60 ± 16.97	125.70 ± 25.55 [#]	134.20 ± 36.79	111.10 ± 28.23
Tryptophan	63.43 ± 14.78	63.50 ± 15.82	67.47 ± 18.04	66.35 ± 19.18	51.67 ± 15.26	53.21 ± 13.27	47.18 ± 10.53	51.92 ± 12.34
Tyrosine	74.18 ± 21.30	66.28 ± 19.90 [#]	71.50 ± 27.78	68.98 ± 23.50	61.45 ± 18.21	63.97 ± 25.91	67.45 ± 25.17	67.97 ± 16.23
Valine	241.50 ± 69.09	225.90 ± 63.62	226.30 ± 54.81	219.70 ± 60.61	208.60 ± 47.47	225.50 ± 59.43	224.40 ± 60.55	177.50 ± 30.29

(mean ± SD, unit: μM)

* p < 0.05 in responder basal level compared to non-responder by Wilcoxon-rank sum test

[#] p < 0.05 in acamprosate treatment compared to responder basal by paired Wilcoxon signed-rank test

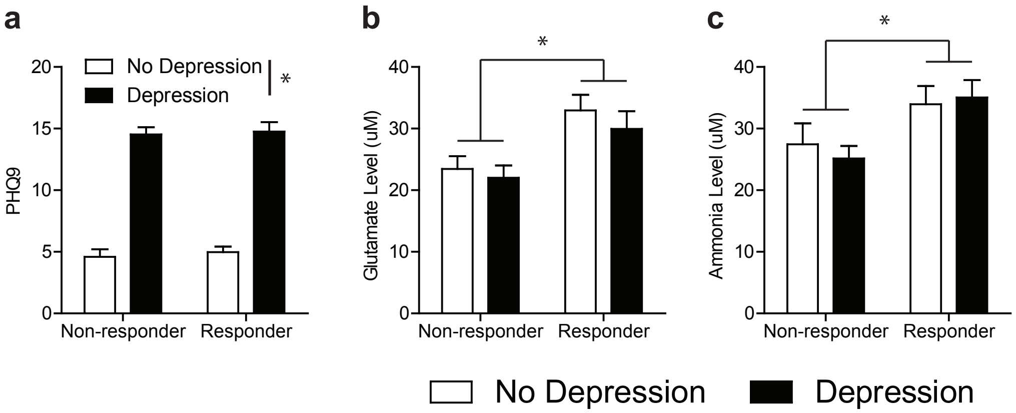

Supplementary Figure 1. The presence or absence of a depression diagnosis does not impact baseline glutamate or ammonia levels when evaluated within response status. (**a**) Responders and non-responders to acamprosate that were diagnosed with depression (responders: *n* = 29; non-responders: *n* = 27) had significantly increased PHQ-9 scores compared to subjects that did not have a depression diagnosis (responders: *n* = 42; non-responders: *n* = 22). *$P < 0.05$ for main effect of depression status. Statistics by two-way ANOVA. Data are reported as mean ± SEM. (**b**) The presence (responders: *n* = 29; non-responders: *n* = 27) or absence (responders: *n* = 42; non-responders: *n* = 22) of a depression diagnosis did not impact the level of baseline glutamate levels indicating that regardless of whether or not a subject was diagnosed with depression, responders to acamprosate exhibited significantly increased glutamate levels compared to non-responders. *$P < 0.05$ for main effect of response status. Statistics by two-way ANOVA. Data are reported as mean ± SEM. (**c**) The presence (responders: *n* = 29; non-responders: *n* = 27) or absence (responders: *n* = 42; non-responders: *n* = 22) of a depression diagnosis did not impact the level of baseline ammonia levels indicating that regardless of whether or not a subject was diagnosed with depression, responders to acamprosate exhibited significantly increased ammonia levels compared to non-responders. *$P < 0.05$ for main effect of response status. Statistics by two-way ANOVA. Data are reported as mean ± SEM.
